# Strategies of Targeting Inflammasome in the Treatment of Systemic Lupus Erythematosus

**DOI:** 10.3389/fimmu.2022.894847

**Published:** 2022-05-18

**Authors:** Yaling Liu, Xinyu Tao, Jinhui Tao

**Affiliations:** ^1^ Department of Rheumatology and Immunology, The First Affiliated Hospital of USTC, Division of Life Sciences and Medicine, University of Science and Technology of China, Hefei, China; ^2^ Department of Clinical Medicine “5 + 3” Integration, The First Clinical College, Anhui Medical University, Hefei, China

**Keywords:** inflammasome, treatment, systemic lupus erythematosus (SLE), target, strategy

## Abstract

Systemic lupus erythematosus (SLE) is an autoimmune disease characterized by multiple organ dysfunction resulting from the production of multiple autoantibodies and adaptive immune system abnormalities involving T and B lymphocytes. In recent years, inflammasomes have been recognized as an important component of innate immunity and have attracted increasing attention because of their pathogenic role in SLE. In short, inflammasomes regulate the abnormal differentiation of immune cells, modulate pathogenic autoantibodies, and participate in organ damage. However, due to the clinical heterogeneity of SLE, the pathogenic roles of inflammasomes are variable, and thus, the efficacy of inflammasome-targeting therapies is uncertain. To provide a foundation for the development of such therapeutic strategies, in this paper, we review the role of different inflammasomes in the pathogenesis of SLE and their correlation with clinical phenotypes and propose some corresponding treatment strategies.

## Introduction

Systemic lupus erythematosus (SLE) is an autoimmune disease characterized by the production of autoantibodies and multisystem damage. The main pathophysiological factors involve adaptive immune system abnormalities, manifested by an imbalance of T cells, excessive B-cell activation, and the production of pathogenic autoantibodies. At present, SLE treatment mainly involves hormonal and immunosuppressant therapies. The resulting immunosuppression is nonspecific, associated with multiple side effects, and its efficacy is uncertain. A better understanding of SLE pathophysiology would help us develop more targeted drug therapies.

Given that abnormal differentiation of T and B cells is critical to the pathogenesis of SLE, most drug development has targeted these cells, their cytokines, and key signal transduction pathways. However clinical translation in large-scale trials has often failed; the main reason for this seems to be that SLE is a clinically and pathologically heterogeneous disease (1). Therefore, a clearer understanding of SLE pathophysiology and more accurate patient phenotyping should result in a more accurate selection of appropriate targeted therapeutic drugs that achieve better clinical outcomes.

Although SLE involves abnormal adaptive immunity, the normal interactions between the innate and adaptive immune systems (2, 3) have attracted interest in how the former is involved in the pathogenesis of SLE. Inflammasomes are oligomeric complexes that make up a critical component of innate immunity as major driving forces for inducing inflammation *via* activation of pathogen-recognition receptors (PRRs). Activated inflammasomes promote the production and secretion of inflammatory cytokines such as IL-1 and IL-18 and play an important role in regulating immune response. Because inflammasomes affect the differentiation of T and B cells (4, 5), they also participate in SLE pathogenesis. In fact, it has been reported that inflammasome-dependent cytokines including IL-1 and IL-18 are involved in the pathogenesis of SLE (6). In patients with SLE, free IL-18 is significantly higher than in controls and is correlated with disease activity (7) related to active kidney disease (8); serum IL-18 can be used as a predictive biomarker for the long-term prognosis of renal function in patients with SLE (9).

SLE is characterized by the production of multiple autoantibodies and multiple organ damage. This is dominated by renal damage and vasculitis. Inhibition of caspase-1 or NLRP3-mediated inflammasome signaling in murine models has demonstrated that inflammasome pathways contribute to SLE pathogenesis by promoting autoantibody production, endothelial dysfunction, and nephritis (10–14). Abnormal activation of the inflammasome was shown to not only be involved in SLE pathogenesis but also be an important risk factor in some SLE patients. It has been reported that inflammasome polymorphisms are associated with susceptibility to SLE (15, 16). Therefore, targeted inflammasome therapy appears to offer a particularly attractive target.

Given the heterogeneity of SLE and a desire for targeted inflammasome inhibition, it will be necessary to select appropriate inflammasome inhibitors for specific SLE patients to achieve maximal efficacy. So far, many proteins that belong to the nucleus binding domain leucine-rich repeat (NLR) family such as NLRP1, NLRP2, NLRP3, NLRP6, NLRP7, NLRP12, NLRC4, and AIM2 (absent in melanoma-2) have been reported to initiate assembling the formation of an inflammasome and in recent years have attracted a lot of attention (17). Each inflammasome is activated in response to different stimuli These inflammasomes play different pathogenic roles. Understanding these roles and their correlation with clinical phenotypes underlies our ability to correctly select matching inhibitors for individual patients. In this review, we will summarize the role and clinical characteristics of the different inflammasomes and put forward a strategy for the targeted inhibition of inflammasomes in the treatment of SLE. We believe that this work will provide an important reference for clinical research and drug development.

## Inflammasomes Are Involved in the Pathogenesis of SLE

Many studies have proposed a pathogenic role for inflammasomes in SLE. Upregulation of inflammasome gene expression and activity has been found in both human and murine lupus (14, 18–21). Due to the interaction between inflammasomes and acquired immunity (22), inflammasomes participate in the pathogenesis of SLE mainly by regulating the abnormal differentiation of immune cells, mediating the pathogenicity of a variety of pathogenic autoantibodies, affecting the pathogenicity of IFN-I, and abnormally activating various signaling pathways including those involved in inflammation. As such, inflammasomes have direct and indirect effects on immune function and thus play a critical role in the pathogenesis of SLE.

### Inflammasomes Regulate the Abnormal Differentiation of Immune Cells

SLE patients have disordered Th1/Th2 and Treg/Th17 balance, which is related to disease activity (23). Inflammasomes help mediate T-cell differentiation. For example, various animal and human studies have shown that NLRP3, NLRP1, NLRC4, and AIM2 may regulate Th1, Tfh, and Th17 cell-mediated immune responses through different pathways, in different immune disorders (24–28). Although there has been no direct evidence showing that inflammasomes mediate T-cell differentiation in SLE, it has been shown that leptin promotes Th17 cell differentiation in lupus erythematosus mice by activating the NLRP3 inflammasome and thus participating in the pathogenesis of SLE (29).

In addition to disordered T-cell balance, abnormal B-cell activation is also critically important in the pathogenesis of SLE. Studies have shown that B cells constitutively express the NLRP3 inflammasome in the cytoplasm, which is activated by typical pathogen-associated molecular products (PAMPs) (30). The expression and activation of NLRP3 in B cells help to maintain B-cell homeostasis and humoral immune responses and are independent of IL-1β participation (31). Given the role of NLRP3 inflammasome in maintaining B-cell homeostasis, it has been suggested that the regulation of B-cell inflammasomes is also an important mechanism in SLE. In addition to NLRP3, it has been found that AIM2 is highly expressed in B cells from SLE patients, promoting B-cell differentiation by regulating the Bcl-6–Blimp-1 axis; this provides a novel target for SLE treatment (32).

Because the primary focus has long been set on the adaptive immune system, the role of neutrophils in SLE-related inflammation has been neglected. However, in recent years, it was discovered that neutrophil extracellular traps (NETs) are critical in driving autoimmune responses (33) and that they are involved in the pathogenesis of SLE (34, 35). In fact, a vicious SLE pathogenic cycle appears to be at play: NETs activate the NLRP3 inflammasome (20) while inflammasomes induce the formation of NETs (36, 37).

### Inflammasomes Mediate the Pathogenicity of Autoantibodies

SLE is characterized by the production of numerous autoantibodies. An interaction between autoantibodies and inflammasomes is involved in the pathogenesis of SLE. As mentioned earlier, inflammasomes are involved in the production of interferon-induced autoantibodies (10), and in turn, autoantibodies produce pathogenicity through inflammasomes.

Production of anti-dsDNA antibodies, diagnosed *via* the antinuclear (ANA) test, is characteristic of SLE and is related to disease activity. In addition to forming immune complexes with dsDNA to produce pathogenicity, anti-dsDNA antibodies also activate the NLRP3 inflammasome and induce human monocytes to secrete IL-1β, triggering a downstream immune cascade and thus participating in the pathogenesis of SLE (18, 19). Anti-dsDNA also promotes lupus nephritis (LN) through the PKCδ–NLRC4 axis (38).

In addition to anti-dsDNA, U1-snRNP antibodies are also important in SLE. It has been found that this antibody activates the NLRP3 inflammasome in monocytes, thereby contributing to the pathogenesis of SLE (39, 40).

### Inflammasomes Affect the Pathogenicity of the IFN-I Pathway

It is recognized that IFN-I plays an important role in SLE (41). Genetic variations of the IFN-I signaling pathway have been shown to increase the risk of SLE in humans and mouse models (42–44).

IFN-I regulates immune responses by activating inflammasomes. For example, in influenza A virus-infected cells, IFN-I signals activate the NLRP3 inflammasome through the TLR3 pathway (45). During streptococcus pneumonia infection, the IFN-I signaling pathway regulates the activation of the AIM2 inflammasome (46). Thus, in SLE induced by IFN-I, inflammasome activation may be an important component of its pathogenicity. Caspase-1 knockout (Casp1^−/−^) mice demonstrate strong protection against prion-induced autoantibody production and IFN-I responses, indicating that caspase-1 is likely to be necessary for IFN-I-induced lupus (10). IFN regulatory factor 1 induced by IFN-I was shown to upregulate inflammasome activity and participate in the pathogenesis of SLE (47).

How IFN-I regulates the inflammasome is incompletely understood and is dependent on the signaling pathway. For example, IFN-I activates the NLRP3 inflammasome through the RIG-I/TLR3 pathway (45), while the STAT1 transcription factor inhibits the activity of the NLRP1 and NLRP3 inflammasomes (48). In addition, the inflammasome itself also affects the expression of IFN-I, mainly playing a negative regulatory role. Studies have found that mice lacking inflammasome sensor AIM2, NLRP3, or adapter caspase-1 produce high levels of IFN-I cytokines, and the activated inflammasome negatively regulates IFN-I signal transduction through MyD88-IRF7 (49).

Therefore, the interaction between the inflammasome and IFN-I is complex, and unexpected results often appear in clinical studies. Clinical studies have shown that expression of the NLRP3/NLRP1 inflammasomes was significantly downregulated in PBMCs obtained from patients with SLE compared with healthy controls; further analysis showed that IFN-I levels were significantly inversely correlated with the expression of NLRP3/NLRP1 inflammasomes, suggesting a negative regulatory effect between IFN-I and the inflammasome in SLE (50).

### Inflammasomes Interact With Various Signaling Pathways

The pathogenesis of SLE is extremely complex and involves many signaling pathways such as NF-KB (51–54), JAK/STAT (55–57), mTOR (58, 59), PI3K/AKT (55), RhoA-ROCK (60), and NETs (34, 35, 61). Direct or indirect evidence has shown that different inflammasomes and various pathways play important roles [(20, 38, 55, 56, 58, 60–99) **Supplementary Table**]. Although most of these findings have not been verified in SLE and the specific mechanisms underlying the interactions have not been made clear, we need to recognize the potential importance of their role. This also highlights the great complexity inherent in the involvement of the inflammasome in the pathogenesis of SLE. For example, NLRP3 (62, 63), NLRP1 (64), and AIM2 (65) inflammasomes are upregulated by NF-KB signaling, while the NF-KB pathway is upregulated by the NLRP3 and NLRP1 inflammasomes (66, 67); these interactions again show the complexity of these mechanisms.

## Different Roles of Inflammasomes in the Pathogenesis of SLE

**Figure 1:**
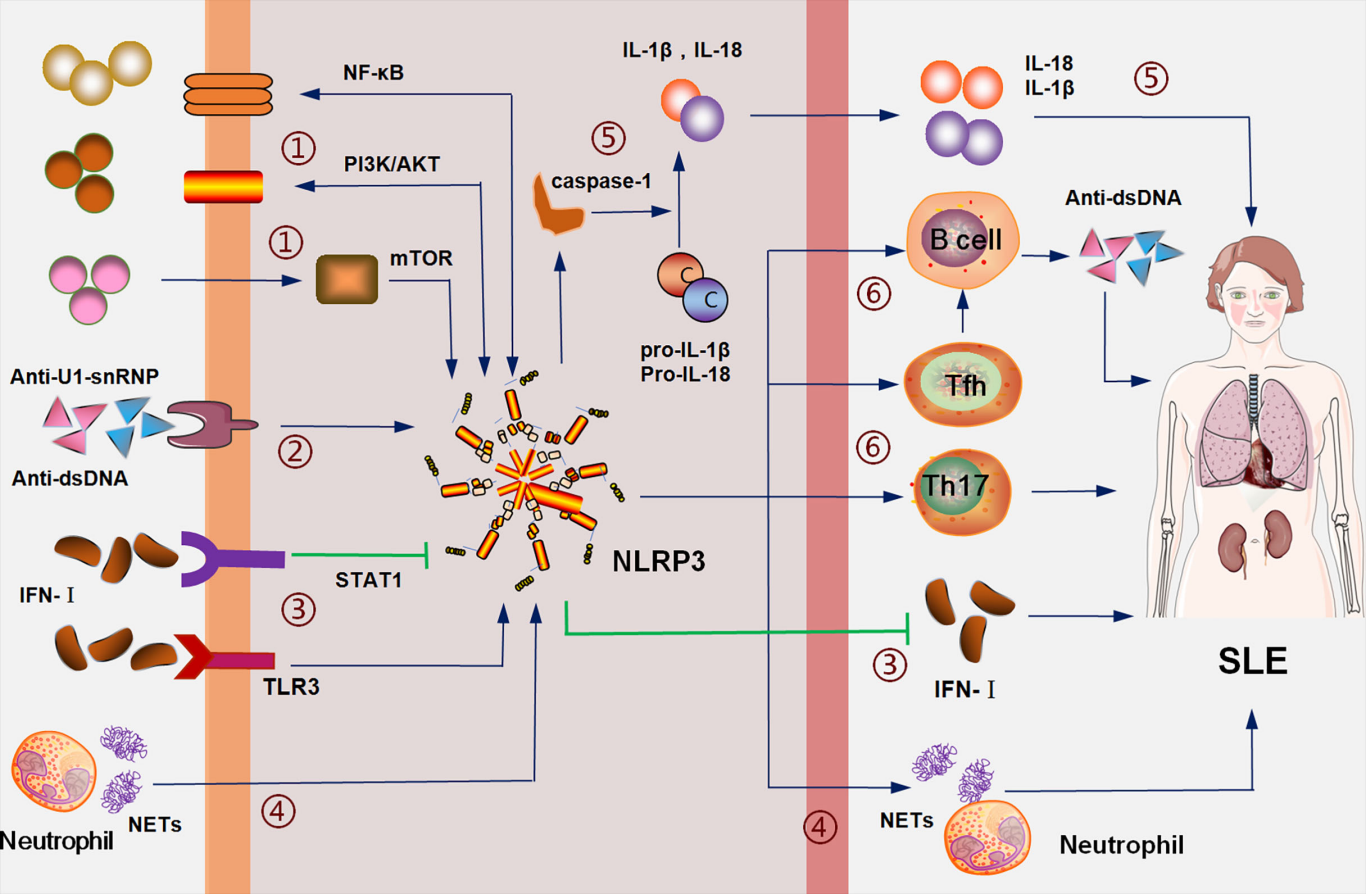
The NLRP3 inflammasome is involved in the pathogenesis of SLE: (1) interacts with SLE pathogenic signals, such as NF-KB, PI3K/AKT and mTOR; (2) mediates the inflammation of pathogenic antibodies including anti-dsDNA; (3) IFN-I activates NLRP3 inflammasome through the TLR3 pathway and inhibits NLRP3 inflammasome through the STAT1 pathway; NLRP3 inhibits the expression of IFN-I; (4) neutrophil NETs activate the NLRP3-associated inflammation, which, in turn, induces the formation of NETs; (5) the NLRP3 inflammasome promotes the maturation and secretion of IL-1β and IL-18 through caspase-1; (6) the NLRP3 inflammasome promotes the proliferation and activation of Th17, Tfh, and B cells to mediate the disorder of cellular immune balance and promote the production of antibodies.

As stated, inflammasomes promote the pathogenesis of SLE through different mechanisms, among which NLRP3 has the most direct evidence. It has been shown that NLRP3 mediates the pathogenicity of autoantibodies and IFN-I, interacts with NF-KB and other pathways to regulate the differentiation of T and B lymphocytes, and participates in the pathogenesis of SLE (**Figure 1**). However, in its interaction with IFN-I, IFN-I exerts different effects on NLRP3 through different signaling pathways (45, 48), and NLRP3 inhibits IFN-I signal transduction (49). Therefore, it is not certain what role NLRP3 plays in SLE patients with highly abnormal IFN-I activation.

In addition, AIM2 has a unique dual role in the pathogenesis of SLE. In terms of the IFN pathway, AIM2 inhibits DNA-induced IFN signals and plays a protective role in SLE. For example, in LN, IFN-I induced AIM2 negatively regulates the IFN-I response (100), suggesting that AIM2 may play a protective role in IFN-induced SLE. However, this effect is affected by p202. Studies have shown that increased expression of p202 inhibits AIM2, thus mediating the pathogenicity of IFN in SLE (101); murine studies have also confirmed that p202 inhibits the activation of the AIM2 inflammasome (102, 103).

The expression of AIM2 and p202 proteins differ by cell type and sex (104, 105); AIM2 is mainly expressed in male patients and innate immune cells, and the p202 protein is mainly expressed in female patients and adaptive immune cells. Therefore, in terms of the impact on IFN signals, AIM2 has stronger pathogenicity in male SLE patients and may be protective in female patients.

Unlike its effect on the IFN signaling pathway, AIM2 promotes SLE through other signaling pathways. AIM2 mediates DNA-induced macrophage functional maturation and SLE pathogenesis (106). AIM2 promotes the Th17 cell contributions to SLE (28) and inhibition of AIM2 expression significantly improves the SLE syndrome in mice (106).

In general, AIM2 promotes SLE. Bioinformatics analyses have found significant differences in the gene expression of the AIM2 inflammasome complex in SLE (107). AIM2 mRNA levels were found to be upregulated in the liver, PBMCs, and the spleen of SLE patients compared with healthy individuals (108). Using the AIM2 inhibitor class C1 decoy oligodeoxynucleotides (ODNs) in lupus-prone (NZB x NZW) F1 mice resulted in a delayed onset of glomerulonephritis and prolonged survival (109).

In addition to NLRP3 and AIM2, the NLRC4 and NLRP1 inflammasomes are also involved in the pathogenesis of SLE. For example, genetic evidence shows that NLRP1 gene polymorphisms are associated with SLE (15, 110) and NLRC4-mediated LN caused by anti-dsDNA antibodies (38). However, the few available relevant studies are unable to clearly explain their exact roles in SLE.

## The Role of Inflammasomes in SLE Organ Damage

**Figure 2:**
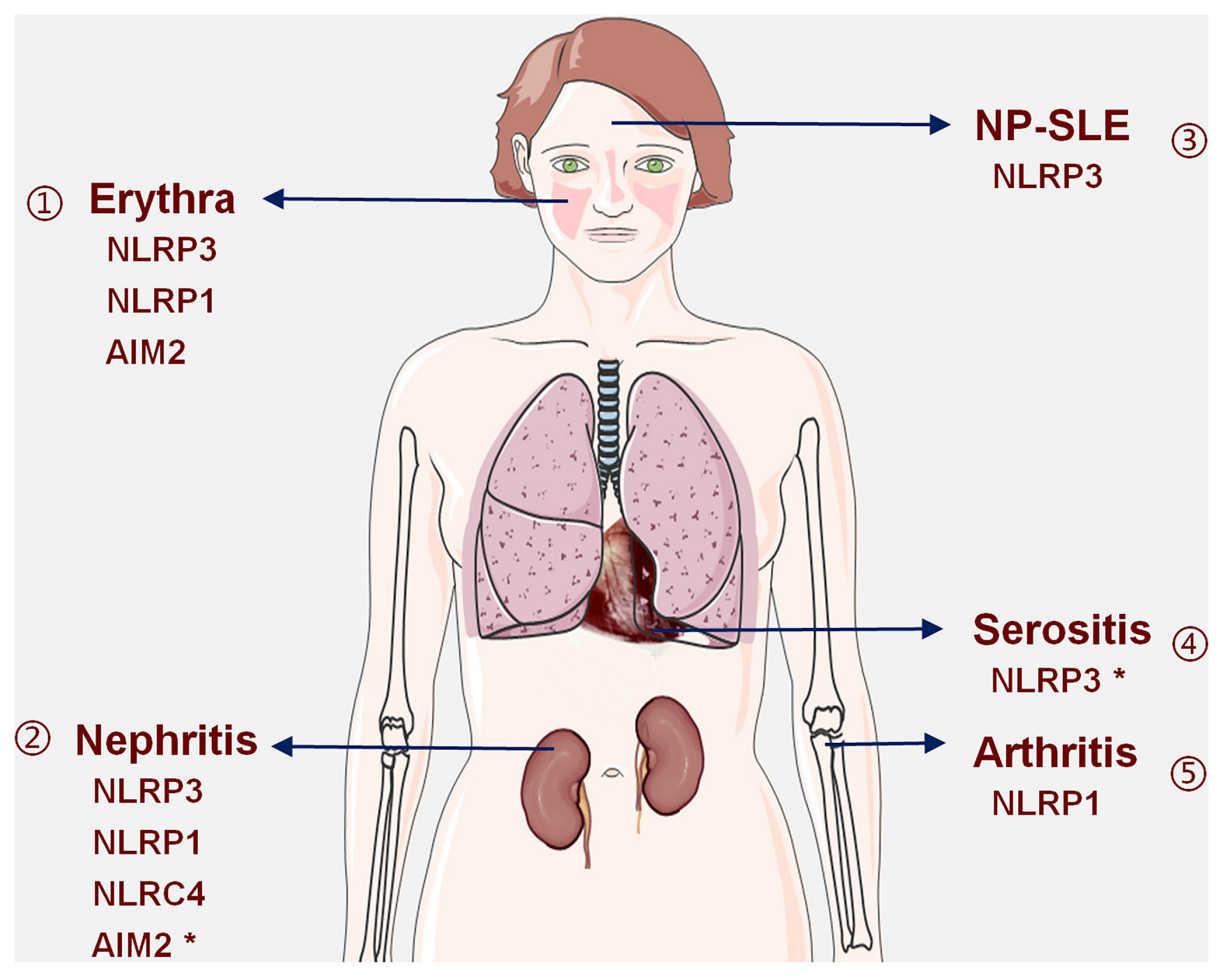
Inflammasomes are involved in SLE-related organ damage: available evidence shows that (1) NLRP3, NLRP1, and AIM2 inflammasomes are involved in erythrina; (2) NLRP3, NLRP1, NLRC4, and AIM2 inflammasomes are involved in LN, and the role of AIM2 inflammasome is complex and may have a protective effect; (3) the NLRP3 inflammasome is involved in the pathogenesis of NP-SLE; (4) the NLRP3 inflammasome may play a promotive or inhibitory role in serositis; (5) the NLRP1 inflammasome is involved in arthritis. *Indicates that this inflammasome has a dual role.

SLE is a heterogeneous autoimmune disease characterized by multiple organ damage. Various inflammasomes are involved in the pathogenesis of SLE through a variety of mechanisms, resulting in different pathophysiologies and clinical manifestations of SLE (**Figure 2**).

### Kidney Damage and Inflammasomes

Kidney damage is the most common organ damage in SLE. As such, it is used as an indication of a successful disease model. Studies have shown that various inflammasomes are involved in this renal damage. For instance, animal experiments have confirmed that activation of the NLRC4 and NLRP3 inflammasomes promotes kidney damage (38, 111–114). Human experiments have also shown that activated NLRP3, NLRP1, and AIM2 inflammasomes are associated with the progression of LN, and that NLRP3 activation has a positive correlation with the activity index (AI) score in LN (115, 116). Moreover, gene expression studies have confirmed that NLRP3 and NLRP1 gene polymorphisms are associated with the development of LN (16, 110, 117) and that the acquired functional variant rs10754558 (NLRP3) is more common in patients with LN, strengthening the view that the NLRP3 inflammasome plays a key role not only in lupus but also in renal damage (16).

Among the many inflammasomes involved in the pathogenesis of LN, the role of AIM2 is particularly uncertain. Human and animal studies have suggested that the AIM2 inflammasome promotes the progression of LN (106). However, from the perspective of the influence of AIM2 on IFN-I signals, AIM2 may play a protective role in the pathogenesis of LN (100). As such, at this stage, it is difficult to explain the role of AIM2 in the pathogenesis of LN.

### Erythema and Inflammasomes

SLE has a strong genetic background; two genetic association studies revealed that variations in IL-1β and NLRP1 are associated with SLE-related erythema in Brazilian cohorts (110, 118). IL-1β is a potent player in cutaneous inflammation and is central in the development of a Th17 micro-milieu in SLE. Studies have also confirmed that the Th17 micro-environment also regulates NLRP1-dependent caspase-5 activity in skin inflammation, suggesting that NLRP1 has an important relationship with skin inflammation (25).

### Serositis and Inflammasomes

Currently, there is inadequate direct evidence to support that inflammasomes cause serositis in SLE. However, previous studies had shown that the 489c > T polymorphism of the P2RX7 gene is associated with the activation of the NLRP3 inflammasome and an increased release of the pro-inflammatory cytokines IL-1β and IL-18; this indirectly reflects that the NLRP3 inflammasome may be involved in the pathogenesis of SLE complicated with pericarditis (119). However, there have also been conflicting findings; the expression and function of P2X7R were shown to be decreased in SLE patients with lupus serositis (120). Therefore, it is uncertain whether serositis is related to the activation of the NLRP3 inflammasome.

### Arthritis and Inflammasomes

Although the role of the inflammasome in the pathogenesis of arthritis has attracted increasing attention (121), their effect on SLE arthritis is not known. At present, it has only been confirmed that NLRP1 gene polymorphisms are associated with SLE arthritis at the genetic level (110).

### Nervous System Damage and Inflammasomes

Neuropsychiatric symptoms of SLE (NP-SLE) are a common manifestation and include psychosis, epileptic seizures, and cognitive dysfunction in severe cases (122, 123). Gene-level studies have shown that NLRP3 gene variations are associated with susceptibility and neurological symptoms of SLE (117). Inhibition of the NLRP3 inflammasome by procyanidin B2 has been shown to be one of the neuropsychiatric symptoms found in the systemic autoimmune MRL-LPR mouse (124), suggesting that the damage to the nervous system may also be related to NLRP3 inflammasome.

## Strategies for Selecting Inflammasome Inhibitors in SLE

**Figure 3:**
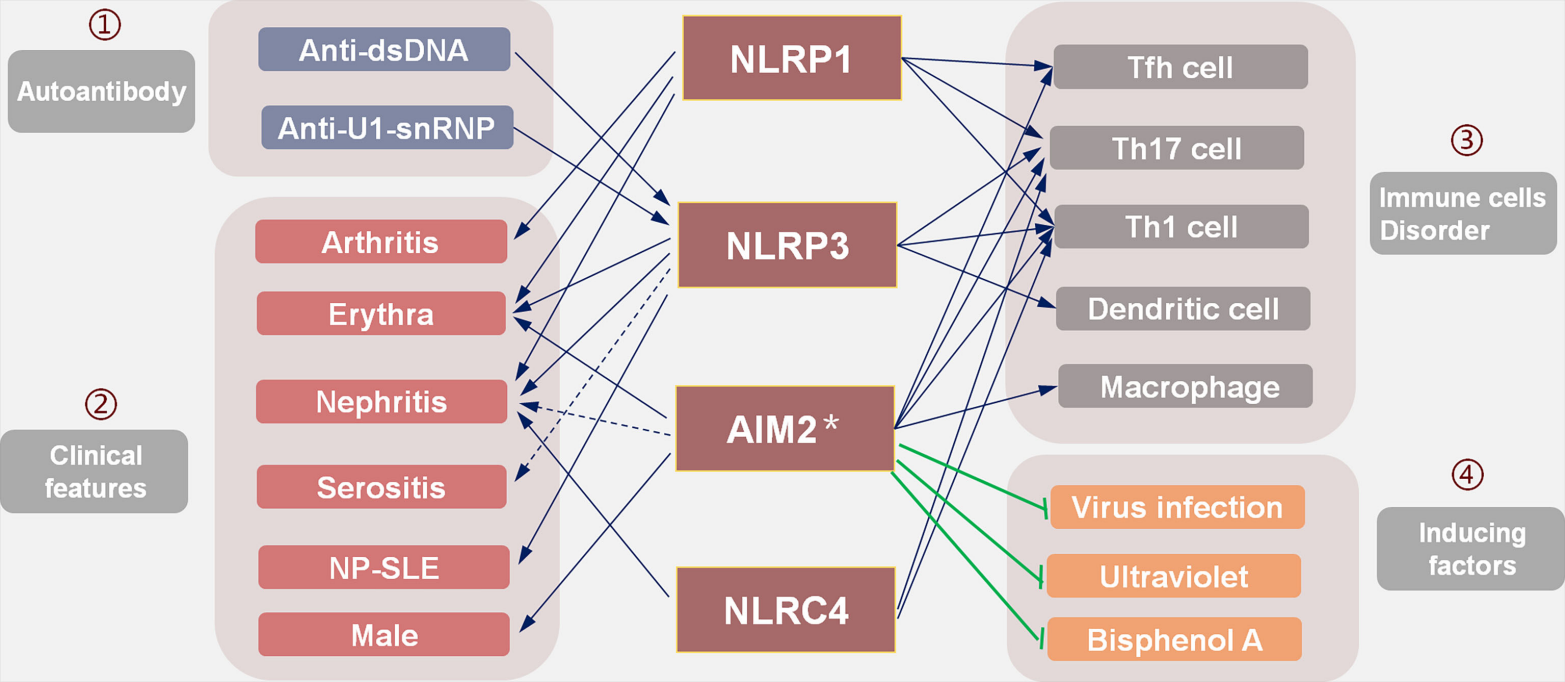
The selection strategies of targeted inflammasome inhibitors in SLE: existing evidence suggests that (1) inflammasomes mediate the pathogenicity of autoantibodies; (2) different organ damage is related to the activation of corresponding inflammasomes; (3) inflammasomes affect the differentiation and proliferation of immune cells and participate in SLE; (4) induced factors such as viral infection, ultraviolet rays, and BPA all play a role through IFN-I, while the AIM2 inflammasome may play a protective role. In the selection of inflammasome inhibitors, many factors should be considered to screen out the inflammasome that plays the main pathogenic role.

Activated inflammasomes promote the production of IL-1 and IL-18, and other inflammatory cytokines, thereby playing an important role in regulating inflammation in SLE. In addition, inflammasomes also interact with many signal pathways related to the pathogenesis of SLE, regulate the differentiation of T, B, and other immune cells, and participate in the pathogenesis of SLE. Therefore, therapeutic inhibition of inflammasomes is a promising direction for precision therapy of SLE. However, there are no clinical, preclinical, or animal studies testing the treatment of SLE with inflammasome inhibitors. Due to the previously outlined disease heterogeneity, the specific inflammasome inhibitors will need to match the underlying pathophysiology. In clinical situations, we will need to comprehensively test which inflammasomes are involved and select the corresponding inhibitors to effect accurate treatment (**Figure 3**). Herein, we propose such a therapeutic approach utilizing the targeted inhibition of inflammasomes.

### Selection of Inflammasome Inhibitors Based on Clinical Characteristics

Different inflammasomes appear to be associated with different organ damage in SLE (**Figure 2**). The NLRP3 inflammasome is involved in erythema, nephritis, NP-SLE, and other organ damage, so inhibition of the NLRP3 inflammasome may have surprising effects. Since specific inflammasomes are related to organ damage, the appropriate inflammasome inhibitor might be selected according to the clinical picture based on organ involvement in the specific patient. For example, an NLRP1 inflammasome inhibitor may be effective in patients with LN complicated by arthritis or erythema; an NLRP3 inflammasome inhibitor may be more effective in LN patients with NP-SLE.

Laboratory tests are helpful in the selection of inflammasome inhibitors. During the onset of SLE, anti-dsDNA antibodies activate the NLRP3 (18, 19) and the NLRC4 inflammasomes (38), while anti-U1 snRNP antibodies activate the NLRP3 inflammasome (39, 40). Therefore, NLRP3 inflammasome inhibitors should be selected when anti-dsDNA and anti-U1 snRNP antibodies are detected in patients.

Although the AIM2 inflammasome is involved in the pathogenesis of SLE, it seems to be harmful only to male patients (104, 105). The AIM2 inflammasome also has a protective effect on the pathogenicity caused by IFN-I (100). Therefore, AIM2 inflammasome inhibitors are likely more suitable for male or non-IFN-I based SLE patients.

### Selection of Inflammasome Inhibitors Based on the Immune Cell Disorder

The strategy of selecting inflammasome inhibitors based on a patient’s clinical presentation is relatively convenient and straightforward in the average clinical setting. However, due to the complexity of SLE, different mechanisms may underlie the same clinical manifestation; SLE involves various immune cell disorders that can be clinically similar. Disordered immune cell function in SLE patients is regulated by the inflammasome. Therefore, the selection of inflammasome inhibitors should be based on the specific immune disorder.

NLRP3, NLRP1, NLRC4, and AIM2 inflammasome inhibitors should be considered in SLE patients who show disordered Th1/Tfh or Th17/Treg profiles; the NLRP3 inhibitors may be helpful for patients with dendritic cell disorders (22). Because the expression of the AIM2 inflammasome is closely related to macrophage activation (106), this inflammasome can promote Th17 cell differentiation (28). Therefore, AIM2 inflammasome inhibitors may be useful in patients with these cell disorders.

### Selection of Inflammasome Inhibitors Based on Inducing Factors

We lack effective methods to comprehensively profile immune abnormalities in clinical settings. However, with an emerging understanding of SLE pathogenesis, the immune abnormalities caused by different inducing factors are becoming clearer. Therefore, understanding the main mechanism involved in the pathogenesis of SLE based on disease-inducing events can also help in the selection of inflammasome inhibitors. For example, virus infection is a common cause of recurrence or aggravation and involves increased IFN levels. It is recommended not to use AIM2 inflammasome inhibitors if there is a clear viral infection, as the AIM2 inflammasome has a protective effect on IFN-I (100). Ultraviolet radiation is a common factor that triggers SLE. Studies have shown that ultraviolet radiation caused the onset of SLE through abnormal activation of IFN-I signaling pathways (125, 126). Likewise, it is recommended not to treat such cases with AIM2 inflammasome inhibitors. In mice and human bone marrow cells, it has been shown that bisphenol A (BPA)-induced signals stimulate IFN-I signaling and further promote the pathogenesis of SLE (127). Therefore, SLE patients exposed to BPA should not use AIM2 inflammasome inhibitors.

## Future Prospects

The inflammasome is involved in the immune mechanism of SLE, but it does not cause SLE. Considering that the inflammasome is positively correlated with disease activity, especially with inflammation related to IL-1 and IL-18, the inflammasome plays an important role in the adaptive immune imbalance. Therefore, inflammasome antagonists could play an important role in controlling disease activity and preventing recurrence.

Due to the paucity of relevant research and the fact that most studies involve animal models, we are yet to fully understand the role and clinical characteristics of inflammasomes in SLE. At present, studies have mainly focused on the NLRP3 inflammasome but this does not dismiss the potential effectiveness of other inflammasome antagonists in the treatment of SLE. The evidence behind inflammasome inhibitor selection is currently rudimentary but intended to serve as a conceptual foundation to stimulate further research in this field. Considering the complexity of SLE pathogenesis, further clinical trials are needed. We also need to continue pre-clinical mechanism-based research in parallel, which will ultimately allow us to identify biomarkers of precision treatments in the clinical–basic–clinical research cycle. In addition, because inflammasomes are activated in many pathways and interact with a variety of pathogenic signals in SLE, basic research is more conducive to obtaining accurate and reliable data for the selection of appropriate animal models (128, 129) and adopting different blocking strategies (128) according to the activation mechanism of the inflammasome.

The selection strategy of targeted inflammasome inhibitors provided in this paper is not perfect, but it provides a clear process for current clinical applications and research. Given the heterogeneity of SLE, most of the targeted therapy drugs do not perform well in clinical trials (1). A selection strategy of inflammasome inhibitors based on specific mechanisms will divide patients into clinical and pathophysiological subtypes, ensuring several uniform pathogenesis groupings that should improve the success rate of clinical trials and increase the efficacy of patient treatments.

## Author Contributions

YL and XT wrote the manuscript. JT contributed to providing the general idea and edited the manuscript. All authors contributed to the article and approved the submitted version.

## Funding

This work was supported by the National Natural Science Foundation of China (81771774) and the Anhui Provincial Key Research and Development Plan (201904a07020103).

## Conflict of Interest

The authors declare that the research was conducted in the absence of any commercial or financial relationships that could be construed as a potential conflict of interest.

## Publisher’s Note

All claims expressed in this article are solely those of the authors and do not necessarily represent those of their affiliated organizations, or those of the publisher, the editors and the reviewers. Any product that may be evaluated in this article, or claim that may be made by its manufacturer, is not guaranteed or endorsed by the publisher.
