# Supplementary material for: Strategies of Targeting Inflammasome in the Treatment of Systemic Lupus Erythematosus
Source: Front Immunol. 2022 May 18;13:894847. doi: 10.3389/fimmu.2022.894847 (PMC9157639; doi:10.3389/fimmu.2022.894847)
Supplement: Supplementary file 1 [file Table_1.docx]

**Supplementary Table** The interaction between inflammasomes and SLE pathogenic signals

| **Signaling pathways promoting the pathogenesis of SLE** | **Interaction between inflammasome and pathogenesis signal of** | | | |
| --- | --- | --- | --- | --- |
|  | **NLRP3** | **NLRP1** | **NLRC4** | **AIM2** |
| JAK/STAT (56, 68) | JAK/STAT pathway activated NLRP3 (69) | **-** | **-** | JAK2/STAT1 pathway activated AIM2 (70) |
| PI3K/AKT (55) | NLRP3 regulated PI3K/AKT pathway (71), vice versa (72, 73) | **-** | **-** | AIM2 inhibited PI3K/AKT pathway (74) |
| RhoA-ROCK (60) | RhoA-ROCK pathway activated NLRP3 (75) | **-** | **-** | **-** |
| NF-κB (76) | NF-κB pathway activated NLRP3 (62, 63), vice versa (66) | NLRP1 inhibited NF-κB pathway (67)  NF-κB pathway activated NLRP1 (64, 65) | **-** | NF-κB pathway activated AIM2 (65) |
| cGAS-STING (77, 78) | cGAS-STING pathway activated NLRP3 (79. 80) | **-** | **-** | AIM2 inhibited STING pathway (81) |
| Notch (82, 83) | Autophagy/Notch pathway activated NLRP3 (84) | **-** | **-** | **-** |
| mTOR (58) | mTOR pathway activated NLRP3 (85) | **-** | mTOR pathway activated NLRC4 (86) | mTOR inhibited AIM2 (87) |
| TLR-MyD88 (88) | TLR-MyD88 pathway activated NLRP3 (62, 89) | **-** | **-** | **-** |
| NETs (61) | NETs pathway activated NLRP3 (20, 90) | **-** | **-** | **-** |
| ERK (91) | ERK pathway activated NLRP3 (92, 93) | **-** | **-** | **-** |
| JNK (91) | JNK pathway activated NLRP3 (94-96) | JNK pathway activated NLRP1 (97,98) | **-** | **-** |
| Pim-1 (99) | Pim-1 pathway activated NLRP3 (99) | **-** | **-** | **-** |
| PKCδ (38) | **-** | **-** | PKC-δ pathway activated NLRC4 (38) | **-** |
